# Delivery Systems for Mitochondrial Gene Therapy: A Review

**DOI:** 10.3390/pharmaceutics15020572

**Published:** 2023-02-08

**Authors:** Rúben Faria, Prisca Boisguérin, Ângela Sousa, Diana Costa

**Affiliations:** 1 CICS-UBI—Health Sciences Research Centre, University of Beira Interior, 6201-506 Covilhã, Portugal; 2 PhyMedExp, Université de Montpellier, INSERM, CNRS, 34295 Montpellier, France

**Keywords:** gene therapy, mitochondria, mitochondria-targeted delivery systems, mitochondrial diseases, mitochondrial gene therapy

## Abstract

Mitochondria are membrane-bound cellular organelles of high relevance responsible for the chemical energy production used in most of the biochemical reactions of cells. Mitochondria have their own genome, the mitochondrial DNA (mtDNA). Inherited solely from the mother, this genome is quite susceptible to mutations, mainly due to the absence of an effective repair system. Mutations in mtDNA are associated with endocrine, metabolic, neurodegenerative diseases, and even cancer. Currently, therapeutic approaches are based on the administration of a set of drugs to alleviate the symptoms of patients suffering from mitochondrial pathologies. Mitochondrial gene therapy emerges as a promising strategy as it deeply focuses on the cause of mitochondrial disorder. The development of suitable mtDNA-based delivery systems to target and transfect mammalian mitochondria represents an exciting field of research, leading to progress in the challenging task of restoring mitochondria’s normal function. This review gathers relevant knowledge on the composition, targeting performance, or release profile of such nanosystems, offering researchers valuable conceptual approaches to follow in their quest for the most suitable vectors to turn mitochondrial gene therapy clinically feasible. Future studies should consider the optimization of mitochondrial genes’ encapsulation, targeting ability, and transfection to mitochondria. Expectedly, this effort will bring bright results, contributing to important hallmarks in mitochondrial gene therapy.

## 1. Introduction

Mitochondria are involved in multiple processes that occur in cells, playing an essential role in maintaining cellular homeostasis [1]. This organelle has its own genome, the mtDNA, that is often affected by genetic mutations, giving rise to mitochondrial dysfunction, and compromising the normal functioning of cells [2]. Nowadays, there are no treatments for diseases originating from mtDNA mutations, and therefore there is an urgent need for the development of new therapies [3]. In this review, we demonstrate how mitochondrial gene therapy can be suitable as a valuable approach to deal with these pathologies. This review aims to present and discuss studies that have explored gene therapy applied to mitochondria and the different types of transporters used to mediate direct mitochondria transfection. A set of delivery systems that have demonstrated the ability to deliver other therapeutic molecules into mitochondria were also presented to elucidate the reader concerning their potential and as a conceptual design to instigate the development of novel and efficient nanocarriers for mitochondrial gene therapy.

### 1.1. Mitochondria

Mitochondria are small organelles present in eukaryotic cells that play a key role in maintaining cellular activity through intracellular signaling and energy production processes. These multifunctional organelles adapt their functioning depending on the cells in which they are present, having specific functions in different cell types [4]. Mitochondria are the engines of cells, as they are responsible for about 90% of the energy that is produced in each cell. Through the mitochondrial oxidative phosphorylation system (OXPHOS), the oxidative phosphorylation of glucose occurs in the mitochondria, which gives rise to energy in the form of adenosine triphosphate (ATP) molecules [5]. In addition to its energetic function, mitochondria are involved in a wide range of biological processes within the cell, namely in amino acid metabolism, protein synthesis, gluconeogenesis, fatty acid oxidation, generation of reactive oxygen species (ROS), ions and calcium homeostasis and initiation of apoptotic cascade [6,7,8]. In addition, mitochondria play a fundamental role in oxidative stress situations, endoplasmic reticulum stress, and stress due to the lack of nutrients that are involved in the origin of DNA and RNA molecules and the processes of transcription correction [9].

Mitochondria have their own DNA allowing their reproduction without the need for outside stimuli. These unique characteristics tend to prove the bacterial origin of this cell organelle. Despite having their own genome, many of the mitochondrial genes were passed to the nucleus, still maintaining a small number of genes that encode essential and exclusive proteins. Among these proteins expressed, only in the mitochondrial matrix, are the four enzymes of the OXPHOS complex [10]. Mitochondria are formed by an outer and an inner membrane in which the inner membrane is folded and forms layers (cristae). They also have an intermembrane space, ribosomes, and genetic material. These organelles, with a size of around 1 micron, can be found in large numbers (1000 to 2000) in the cytoplasm of eukaryotic cells [11].

### 1.2. Mitochondrial Genome

The mitochondrial genome is made up of circular double-stranded DNA molecules, a characteristic indicating the bacterial origin of mitochondria [12]. Mitochondria evolved from proteobacteria, and throughout this evolution, these organelles had significant changes in their genome, where there was a significant gain of new genes and the loss and transfer of others to the nuclear genome. This transition led to changes in the mitochondrial proteome and the development of additional roles in both the metabolism and biosynthetic pathways, forming a specialized organelle for ATP production [13,14].

The human mitochondrial genome is made up of only 37 genes that encode 13 mRNAs, which in turn are translated into 13 proteins. It also has 22 tRNA and 2 rRNA molecules (12S and 16S) in its constitution [15]. However, to control the expression of mitochondrial genes, the intervention of proteins originating in the nucleus is also necessary, which causes this regulation to have two origins, namely mitochondrial RNAs and nucleus proteins. Most of the proteins necessary for the functioning of the mitochondria are encoded by the nucleus, being later synthesized in the cytoplasm, and imported into the mitochondria. To control their energy metabolism, mitochondria regulate the synthesis of their RNAs, which in turn determine the steady state of mitochondrial proteins [16,17].

**Figure 1:**
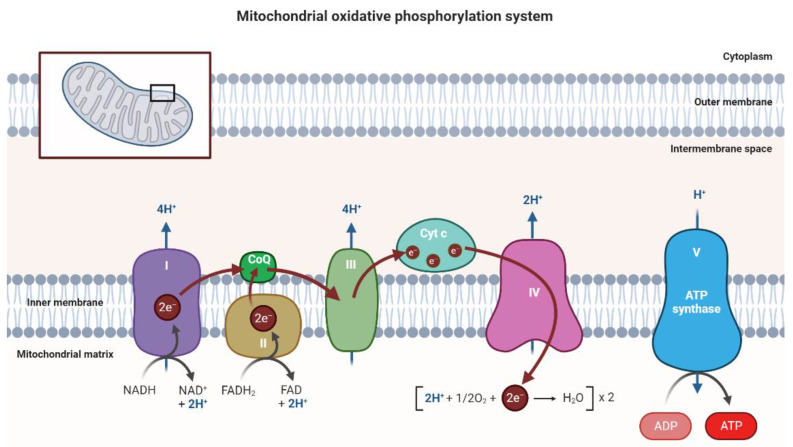
Schematic representation of the OXPHOS system composed of five large complexes and functional proteins such as CoQ and Cyt c located in the inner mitochondrial membrane, where the flow of electrons and protons through the respiratory chain is observed and where ATP synthesis occurs. Image created at BioRender.com.

Each strand of double-stranded mtDNA has a different composition. The lighter chain only contains the information to transcribe 8 tRNAs and a polypeptide while the guanine-rich chain contains information to encode 14 tRNAs, 2 rRNAs, and 13 polypeptides [18]. The 13 polypeptides encoded by mtDNA give rise to 11 polypeptide constituents of the electron transport system (ETS) and 2 polypeptides of the ATPase complex [16,19]. In the NADH-ubiquinone reductase complex (complex I), there are 7 polypeptides encoded by the mitochondrial genome (subunits ND1, ND2, ND3, ND4, ND4L, ND5, and ND6). In the succinate dehydrogenase complex (complex II) there are 4 subunits encoded by nuclear genes (SDHA, SDHB, SDHC, and SDHD). In the ubiquinol–cytochrome c oxidoreductase (complex III), only cytochrome b is encoded by mtDNA [19]. The Cytochrome-C oxidase complex (complex IV) consists of 3 mtDNA encoded subunits (MT-CO1, MT-CO2 and MT-CO3) [20]. The ATP synthase complex (complex V) has 2 subunits (ATP6, ATP8) issued from mitochondrial genes [21]. The OXPHOS system has ATP and water as final products, where the oxidation of NADH and FADH2 will generate electrons that are transferred through the respiratory chain (complexes I-IV) to oxygen. This transfer creates an electrochemical gradient that is used by ATP synthase to generate ATP molecules [22]. The OXPHOS system is represented in Figure 1.

The mtDNA also has a region called the regulatory region, a non-coding region that is essential for the transcription of the genome and its replication. In this non-coding part, L-and-H strand promoters (ITL and ITH, respectively) and two putative origins of replication (OH and OL) are present, from which mitochondrial transcription is initiated. This transcription process is carried out by the DNA-dependent RNA polymerase where the respective cofactors must be present [23].

The fact that mtDNA lacks telomeres and lacks introns makes it more susceptible to mutations when compared to the nuclear genome [12]. Alterations that compromise the normal expression of mtDNA can result in a multisystemic disease phenotype derived from the disturbance of the respiratory chain, mainly affecting neuronal and muscular tissues [16]. In recent years, several studies have shown that biochemical and genetic changes in mitochondria lead to the emergence of multisystemic diseases [24]. Research in animals has revealed that mitochondrial mutations have a direct influence on the immune response [25], metabolic regulation [26], brain function [27], and the aging rate and lifespan [28,29].

### 1.3. Mitochondrial Mutations and Associated Diseases

The full understanding of the origin of many mitochondrial diseases remains a mystery today. However, it is known that deregulated mechanisms and interactions will unbalance normal mitochondrial functioning. The maintenance of mitochondrial function depends on several processes, namely the correct assembly of the OXPHOS system as well as the regulation and control of protein importation and degradation in general. The coordinated interaction between the mitochondrial and the nuclear genome, the mechanisms for controlling replication, transcription, translation, and the correct functioning of DNA repair systems are other essential processes. Thus, these mechanisms are potential targets to understand the origin of unknown mitochondrial dysfunctions [2].

Metabolic disorders resulting from changes that occur in genes regulating mitochondrial function are called primary mitochondrial diseases (PMDs) [30]. PMDs are characterized by having different origins and depending on the affected gene can result in multiple metabolic disorders. These disturbances in mitochondrial function and structure will compromise its normal activity, namely the processes of oxidative phosphorylation, mitochondrial fission and fusion, and the process of ion transport across the mitochondrial membrane. Both mtDNA and nuclear DNA mutations can give rise to PMDs and cause mitochondria-associated metabolic changes [31]. Due to the presence of mitochondria in almost every type of organ in the human body, PMDs rarely involve just a single tissue. PMDs can affect many organs and the age factor does not seem to have an influence [32].

Mutations in mtDNA occur at a much higher frequency than mutations in nuclear DNA. These mutations result from errors during replication, and these errors often remain uncorrected. Mutations in mtDNA can also be transmitted by maternal mtDNA, arise from antecedent mutations in nuclear DNA, or result from environmental factors [33]. One of the cases of environmental factors is stress which causes overexpression of reactive oxygen species (ROS). ROS are produced during the process of oxidative phosphorylation by the OXPHOS system, however, their production is at low levels and their presence is fundamental in physiological functions. Increased ROS levels can cause numerous mutations in mtDNA since they are produced within the mitochondria with high proximity to the genetic material [34]. The fact that mtDNA lacks protective histones also makes it more susceptible to mutations caused by ROS [35]. Elevated levels of ROS in situations where antioxidant enzymes are reduced can result in changes in both proteins and lipids and in the mtDNA itself, leading to the appearance of mitochondrial dysfunction. In extreme cases, mutations caused by ROS can alter the OXPHOS system and lead to the production of even more ROS, causing successive changes in mitochondrial metabolism and inducing a decrease in ATP production, loss of cell communication, rupture of the mitochondrial membrane, and consequent apoptosis [36].

Other recurrent factors for mitochondrial deregulation are the mutations that occur simultaneously in the mitochondrial genome and in genes of nuclear origin that influence mitochondrial metabolism. These mutations, when they appear in genes encoding proteins essential for the synthesis of ATP molecules, lead to the degradation of cells due to lack of energy and, consequently, contribute to the appearance of diseases of mitochondrial origin in the various organs of the human body. These mutations can also interfere with the normal functioning of the OXPHOS system, alter tissue specificities of different organs and alter metabolite homeostasis [37].

The heteroplasmy occurs with high frequency in mitochondria and plays a crucial role in the heterogeneity of clinical manifestations that mtDNA mutations can cause. Mitochondrial heteroplasmy is the name given to the coexistence of mutated mtDNA with wild-type mtDNA in the same cell [38]. This coexistence arises due to the high rate of mutations that occur during mtDNA replication, which together with the fact that mtDNA replicates independently of the cell cycle, leads to the replication of thousands of mtDNA molecules with somatic changes. The fact that mtDNA can be segregated during replication also contributes to mitochondrial heteroplasmy, causing levels of heteroplasmy that change from cell to cell and throughout the lifespan in mitotic and post-mitotic cells [39]. Heteroplasmy levels are critical for maintaining homeostasis in mitochondria. Increased heteroplasmy leads to the onset and worsening of mitochondrial dysfunction, contributing to the loss of regulatory mechanisms present in mitochondria that interfere with cell metabolism and signaling pathways. Furthermore, high levels of heteroplasmy lead to the accumulation of depolarized mitochondria and high ROS production [40]. Thus, it can be stated that the onset and/or severity of mitochondrial diseases is dependent on the type and number of mutations and the rate of heteroplasmy present in the cells. Clinical manifestations appear when a certain percentage of mutated mtDNA is reached (between 60 and 90%), and the threshold may vary with the type of cell, tissue type, or type of mutation. Mitotic and meiotic cell division can also alter the levels of heteroplasmy in cells, which means that the speed with which phenotypic changes appear depends on the ability of the cell or tissue to replicate [39].

Pathologies originating in the mitochondria arise, in most cases, from mutations that are inherited from the mother, resulting in dysfunctions in oxidative energy metabolism. However, there are also somatic mutations in mtDNA, and these alterations in the mitochondrial genome are at the origin of aging, neurodegenerative diseases, and cancer [41]. One of the changes that most contributes to the appearance of disturbances in the normal functioning of mitochondria are point mutations. This type of mutation consists of alterations of nitrogenous bases in mtDNA genes (tRNALeu(UUR), tRNALys, and ATP6 are the most common) and that give rise to diseases such as mitochondrial encephalopathy, stroke-like episodes (MELAS), myoclonus epilepsy, and ragged red fibers (MERRF) syndrome [41]. Beyond point mutations, mtDNA deletions can occur, which result in a loss of part of the mitochondrial genome. The elimination of essential genetic information leads to the appearance of Pearson’s syndrome, Kearns Sayre syndrome, and chronic progressive external ophthalmoplegia. This type of mtDNA change has no maternal origin and appears randomly in mtDNA molecules, which means that the mutation occurs in early embryogenesis or the germ line of the mother. The incidence of point mutations in mtDNA occurs more frequently in mitotic cells, whereas deletion mutations appear to a greater extent in postmitotic cells [42].

**Figure 2:**
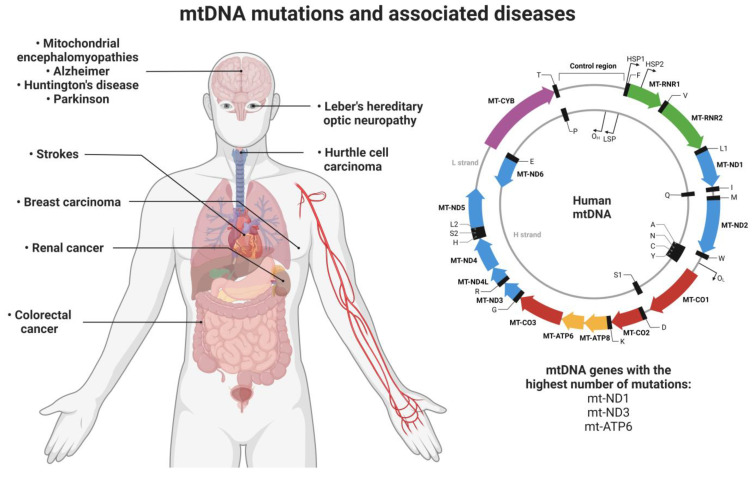
Illustration of the mitochondrial genome with a focus on the mitochondrial genes that suffer the greatest number of mutations (ND1, ND3, and ATP6) and the organs/tissues affected by these mutations and their associated diseases. Image created at BioRender.com.

Several mutations in mitochondrial genes trigger the appearance of pathologies (Figure 2). In the case of type II diabetes, mutations in the mitochondrial genes ND1, ND3, and ATP6 are reported to be associated with this disease [43,44,45]. Mutations in the ND1 gene are also associated with the onset of Leber’s hereditary optic neuropathy (LHON) syndrome [46,47]. In the work published by Smolina et al., six mutations in the Desmin gene (DES) that are associated with skeletal and Desmin myopathies were identified [48]. Mitochondrial encephalomyopathies are also associated with a mutation in the ATP6 gene [49]. In neurodegenerative diseases, mitochondrial mutations are also found to contribute to the onset of diseases. For example, in amyotrophic lateral sclerosis and associated frontotemporal dementia, mutations in the C9orf72/TARDBP genes have been identified [50]. Huntington’s, Alzheimer’s, and Parkinson’s diseases are other neurodegenerative pathologies associated with mitochondrial dysfunction [51]. In the case of Parkinson’s, a mutation (A10398G) was identified in the ND3 gene, while in Alzheimer’s disease, there is a mutation in the mitochondrial ATP6 gene [52,53]. Mutations in mtDNA can also cause myoclonic epilepsy with irregular red fibers (MERRF) [54], strokes and encephalopathies (MELAS syndrome) [55], obesity [56], and are also involved in the onset of cancer [57].

In recent years, a huge number of mutations in mtDNA have been discovered that are associated with the appearance of different types of cancer. For example, Ganly et al. reported mutations in virtually every mtDNA protein-coding gene, mutations identified in patients with Hurthle cell carcinoma [58]. Hurthle cell carcinoma is a type of tumor within the thyroid gland, and its appearance is associated with mutations in mtDNA, namely mutations in the ATP6 gene (Atp6-K90E) and deletions in several genes of complex I of the respiratory chain [59,60]. In cancers of the digestive system, four mutations were identified in the mtDNA genome (11719A/12705T/15043A/15301A) [61], while in invasive lobular breast carcinoma, a mutation was identified in the mitochondrial gene COX3 [62] and in breast cancer, the mutation “8584G>A” was identified in the gene ATP6 [63]. The mitochondrial gene ND1 is involved in several types of cancer when it undergoes a mutation identified as “C3572ins”, namely in renal cancer, thyroid carcinoma, oncocytic pituitary adenoma, and invasive ductal breast carcinoma [57,62,64,65]. The mutated mitochondrial genes and their associated diseases are summarized in Table 1.

### 1.4. Conventional Treatments for Mitochondrial Dysfunctions

Recent research has shown that mtDNA mutations are present in one in 250 people with at least one in 5000 suffering from severe disease. Currently, the drugs available on the market for mitochondrial diseases are very limited and only serve to mitigate the symptoms [69]. Of all the FDA-approved options and the multiple strategies that have been studied in recent years, no drug has managed to provide a cure or slow the progression of mitochondrial diseases. Clinical trials in recent years aimed at the development of new drugs for the treatment of mitochondrial diseases do not exceed 100 and very few reach Phase III. The ones reaching stage III demonstrate the potential to improve a patient’s quality of life by relieving symptoms caused by changes in mitochondria, but none can provide an effective cure [70].

The complexity of mitochondrial disorders and the heterogeneity of clinical manifestations lead to several approaches for the development of new mitochondrial therapies. One of the strategies studied is the use of antioxidants to reduce the toxicity caused by high levels of ROS that are formed in certain mitochondrial diseases. One of the strategies is the use of lipoic acid, a cofactor in several dehydrogenases, including pyruvate dehydrogenase, branched-chain amino acid dehydrogenase, and α-ketoglutarate dehydrogenase [71]. Lipoic acid is administered together with other antioxidants such as CoQ10 and creatine monohydrate, demonstrating the ability to decrease the levels of oxidative stress markers and muscle strength restoration in individuals diagnosed with mitochondrial diseases [72,73]. Regulation of ROS levels can also be achieved indirectly, by administering cysteine to patients in whom glutathione levels are decreased due to mitochondrial disturbances. Glutathione is an antioxidant present inside cells whose synthesis depends on the presence of cysteine [74]. Another approach using vitamins C and E as antioxidants was tested in conjunction with other drugs but did not show promising results [74].

In patients with myopathy and mitochondrial encephalopathy, creatine monohydrate is administered to improve their quality of life. Creatine in the presence of phosphate creates phosphocreatine which is essential in the process of releasing energy in tissues that need high amounts of energy such as the brain and muscles. In these patients, phosphocreatine levels are compromised, and the administration of creatine monohydrate improves motor skills and brain function [75,76]. In patients diagnosed with dilated cardiomyopathy and skeletal myopathy, there is a change in the functioning of the mitochondrial respiratory chain and consequently in the production of ATP [77]. In these cases, the use of the synthetic peptide MTP-131 makes it possible to regulate the functioning of cardiolipin, a phospholipid essential for the structure of the inner membrane of the mitochondria and the correct functioning of the respiratory system [78,79]. The binding of MTP-131 to cardiolipin improves the flow of electrons in the electron transport chain, which could lead to an increase in ATP production in these patients [80]. The benzoquinone idebenone compound (2,3-dimethoxy-5-methyl-6-(10-hydroxydecyl)-1,4-benzoquinone) was used in LHON patients and its administration was shown to be effective in protecting patient’s eyesight. The use of idebenone prevented the development of dyschromatopsia and the loss of the ability to see colors in patients with LHON [81].

The examples presented, among others available in the literature [82,83,84], demonstrate the inefficacy of the currently available drugs for the treatment of mitochondrial diseases. Therefore, there is an urgent need for more effective treatments/approaches that may solve the problem at the root, bringing hope to the millions of patients worldwide suffering from mitochondrial disorders. One of the approaches that can be explored is the use of mitochondrial gene therapy since it allows one to act directly on the origin of the problem and develop new personalized therapies for each case, through the use of therapeutic genetic material.

## 2. Nanotechnology in Mitochondrial Gene Therapy

### 2.1. Mitochondrial Gene Therapy

The development of nanotechnology has allowed the conception/emergence of new therapies, one of them being gene therapy. Gene therapy is based on the use of recombinant DNA techniques with functional genes to replace defective genes and consequently treat associated diseases [85]. This type of therapy can be used to especially treat diseases originating in monogenetic changes or when mutations are well identified [86]. Many of the dysfunctions that occur in mitochondria come from mutations in their genome, thus gene therapy emerges as a very promising approach to the treatment of mitochondrial diseases. Mitochondrial gene therapy arises from the need to find treatments for mitochondrial dysfunctions, as the solutions available on the market only serve to alleviate the symptoms and do not provide an effective cure [87]. The main advantage of this approach, to conventional treatments, is that it focuses the problem on its origin replacing the mutated mitochondrial gene and restoring mitochondria function. In addition, mitochondrial gene therapy is a technique with reduced costs that can provide continuous treatment in time on target cells [88].

Although most mitochondrial diseases in adulthood result from mtDNA mutations, alterations in nuclear genes that have a direct influence on mitochondrial metabolism also induce this type of disease, being the main cause of mitochondrial dysfunctions in children [30,89]. Thus, nucleus targeting has been explored to correct mitochondrial disorders. The application of indirect mitochondrial gene therapy aims at the translation of a protein originating from the genes transferred to the nucleus that is later imported into the mitochondria, reestablishing its normal function. One example is mitochondrial dysfunction cardiomyopathy, where a mutation in the adenine nucleotide translocator 1 gene alters a protein in the inner membrane of the mitochondria and affects the exchange of ADP and ATP [90]. Reyes et al. demonstrated that a mutation in the RNASEH1 gene compromises mtDNA replication, affecting several complexes of the respiratory chain and consequently the metabolic activity of mitochondria. This mutation affects mtDNA integrity and can lead to chronic progressive external ophthalmoplegia [91].

Gene therapy requires exogenous DNA to reach target cells. Physical, chemical, and biological methods have been explored in recent years to assess which approach is more convenient for each situation. Physical methods were the first approaches considered for the delivery of genetic material. These methods do not use carrier molecules for gene delivery. The advantages of these techniques are that transfection is not dependent on the ability of transporters to internalize cells and, therefore, there are no biocompatibility concerns related to the materials used in the conception of the delivery vectors. Despite this, physical methods can destroy the membrane of target cells as they are more invasive [92]. In this regard, the most used techniques are microinjections and bioballistics. Microinjections consist of introducing genetic material with the help of a micropipette; however, this procedure requires a lot of experience and technique to avoid the bursting of the cell membrane. The bioballistic technique uses air pressure to project complexes of nucleic acids that are normally coated with metals [93]. Other physical methods are emerging to overcome the disadvantages of these invasive techniques, which are the case of electroporation [94], optoporation [95], sonoporation [96], and magnetoporation technique [97]. These techniques aim to internalize the exogenous genetic material in cells through physical forces, being much less invasive and for many of them there is no contact with the cell membrane [92].

Physical methods have been used for mitochondrial gene therapy as described by Bonnefoy and Fox [98]. These researchers demonstrated the use of a helium shock wave to accelerate metal particles coated with DNA for the internalization in Saccharomyces cerevisiae cells. A low percentage of cells that survived this bioballistic technique was shown to have taken up exogenous DNA [98]. The use of injections as a technique for mitochondrial gene therapy was analyzed by Yasuzaki et al. By creating an artificial mtDNA plasmid DNA with a mitochondrial promoter and a reporter gene, Yasuzaki demonstrated that it is possible to deliver genes without the interference of any carrier molecule. The plasmid was injected directly into rat liver mitochondria in vivo by hydrodynamic injection and the exogenous mRNA was identified by PCR technique. Afterward, the corresponding reporter protein was expressed in the liver and skeletal muscle [99].

**Figure 3:**
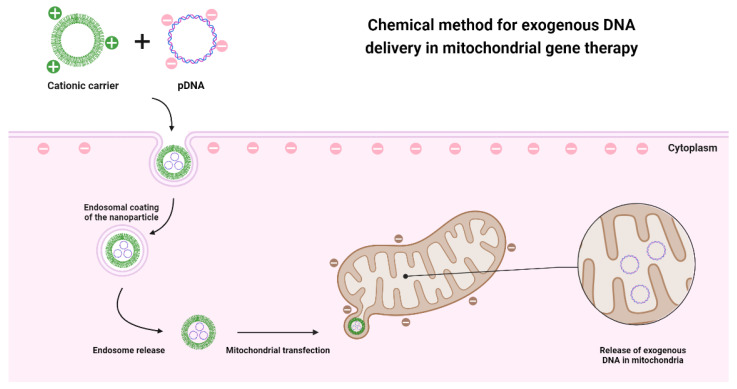
Representative illustration of the application of chemical methods in the delivery of genetic material to the mitochondria, based on the use of a cationic nanotransporter that through electrostatic interactions manages to penetrate both the cell membrane and the mitochondrial membranes.

Although physical methods have some ability to internalize DNA into mitochondria, the success rate is limited. This fact instigates the development of other approaches that could bypass mitochondrial membranes. The most promising and most explored method is the chemical one since it meets the characteristics of mitochondrial and cell membranes. This method requires a cationic carrier that allows exogenous DNA to pass through the membranes, as both entities are negatively charged and would repel each other (Figure 3). Before reaching the outer membrane of the mitochondria, the developed systems must be able to bypass the cell membrane and/or the endosomes after an endocytosis-dependent internalization. Another aspect to consider is, that mitochondrial membranes are hydrophobic, so the carrier molecules must have amphiphilic properties to penetrate the membranes [100].

One of the fundamental steps for this therapy is the choice of the nanocarrier that enables the delivery of therapeutic DNA. The characteristics to consider when choosing the vector are, mainly, its production on a large scale, its biocompatibility, and the ability to carry DNA. Since mitochondria have specific receptors and have a double membrane that is impermeable to hydrophilic molecules, there is a need to create new approaches and specific therapeutic vectors for mitochondrial gene therapy [88,100]. Due to the difficulty in targeting delivery systems to mitochondria, it is convenient to explore their receptors to facilitate their uptake by this organelle. It was found that mitochondrial proteins that are expressed in the cytosol enter the mitochondria through the translocase of the mitochondrial outer membrane (TOM) and mitochondrial inner membrane (TIM) allowing their recognition due to a sequence at the *N*-terminus of these proteins [101]. The presence of the mitochondrial targeting signal peptide (MTS) sequence allows preferential recognition of the TOM20—TOM22 receptor and thus translocation of proteins across mitochondrial membranes [102]. Therefore, the MTS sequence has been used as a ligand in mitochondrial gene therapy, mediated by delivery systems for a targeted delivery [103,104]. Due to the characteristics of mitochondria, gene therapy targeting this organelle is even more challenging than nuclear gene therapy since it has a double membrane and very specific receptors. In this sense, it is necessary to find delivery systems that can internalize the cell and, at the same time, provide specificity and targeting to mitochondria after they reach the cytoplasm. Nanotechnology has allowed the development of new transporters that can be used to deliver therapeutic genetic material to mitochondria.

### 2.2. Nanotechnology

Considered one of the most promising technologies of this century, nanotechnology allows the development of nanoscale materials for applications in the most diverse aspects of society using nanoscience as a theory. It allows the creation of technology at the nanometer scale which finds applications in areas such as engineering, electronics, physics, chemistry, biology, and medicine. Nanotechnology, by definition, deals with the design/development and application of materials with sizes between 1 and 100 nm. It also intends to tailor and optimize the physicochemical properties of these materials, since they can influence their interactions and functionalities [105].

Nanotechnology has become a major object of study in the development of diagnostic methods and new therapies in the field of biomedicine. The application of this technology in the field of biology has enabled the development of novel and advanced therapies, new methods of drug delivery, and new diagnostic tools using molecular imaging [106]. Over the past decades, nanotechnology has given rise to promising and innovative results, and it is not surprising that there are currently numerous nanomaterials already commercialized for medical purposes. The range of commercialized nanopharmaceuticals includes nanoparticles for the delivery of therapeutic drugs, such as anticancer drugs, nanostructures for application in bone restructuring and with antibacterial activity, and nanomaterials for the detection of biomarkers (nanosensors, nanoelectrodes, and nanochips) [107].

Inevitably, nanotechnology has been extensively explored in the field of oncology, where its assets have been explored to develop new treatment methodologies and to improve the effectiveness of drugs in conventional chemotherapy. The application of nanoparticles allowed the development of more targeted therapies for tumors, using functionalized molecules. These nanoparticles can be used as direct therapeutic agents or to serve as carriers of therapeutic biomolecules with anticancer activity [108,109]. Additionally, nanotechnology has been applied to perform diagnosis simultaneously with the therapeutic function. The principle of nanotheranostics is the development of a nanosystem to provide a desired therapeutic effect and, at the same time, allow the visualization of the tissue or cells targeted by this therapy [110]. This technology can, for example, monitor the drug release and its biodistribution as well as evaluate at the same time the therapeutic effect of the drug delivered by the nanosystem. These nanoscale systems also ensure a targeted delivery to specific cells or tissues, using ligands that will be recognized by the receptors of the target cells, providing a more personalized and targeted therapy [111].

Nanotechnology can also be used to identify biomarkers associated with mitochondria and explore them as a method of detecting mitochondrial disorders and early diagnosis. This technology can use mitochondrial biomarkers associated with specific mutations and thus combine them with gene therapy to develop new therapeutic strategies [112]. Another approach is the application of mtDNA itself as a biomarker to identify possible changes and pathologies and to assess the body’s response to drug dosages, particularly in chemotherapy [113]. The identified mitochondrial biomarkers can then be used in gene therapy as therapeutic targets of diseases and enable the intervention in processes such as mitophagy, post-transcriptional regulation, modification of mitochondria, and interactions of this organelle with other cellular organelles [114].

The ability to use nanomaterials as carriers is also explored for gene delivery. One of the most explored applications is the combination of nucleic acids with nanotechnology, formulating nanostructures of reduced size. The use of nanomaterials allows the formulation of nanoparticles with sizes around 100 nanometers, which confers a fundamental physical property for the delivery of genetic material since the size of mitochondria varies between 0.5 and 1µm in diameter and 1µm in length. These nanocarriers are thus an excellent approach for mitochondrial gene therapy [4,115]. These DNA or RNA nanostructures can be applied in diagnostic protocols, determination of the structure of biomolecules, formulation of nanoparticles, and in the areas of synthetic biology and biophysics. Polymers, lipids, and micelles are mostly used in this DNA nanotechnology [116]. This technology has led to emergent areas of study, namely, gene therapy.

### 2.3. Delivery Systems in Mitochondrial Gene Therapy

#### 2.3.1. Polymers

**Figure 4:**
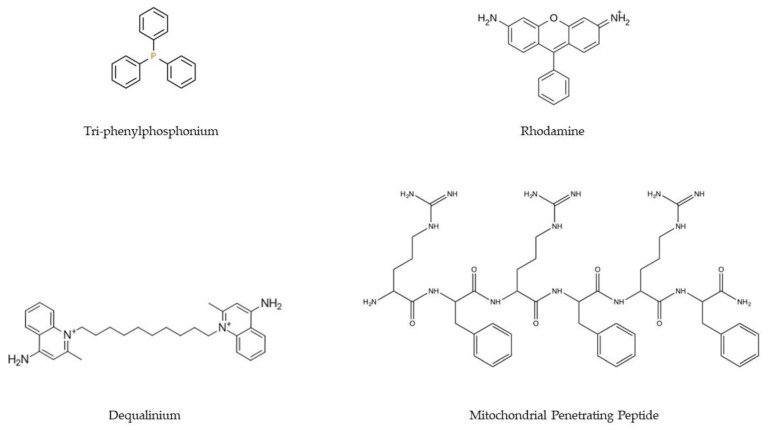
Chemical structures of the main ligands (TPP, rhodamine, DQA, and MPP) used in the development of delivery systems to confer targeting to the mitochondria.

Polymers are one of the most used materials to formulate nanocarriers. Their properties, such as easy manipulation of their structure and composition, ability to incorporate ligands, fast and economical production, and biocompatibility, make this type of material one of the most explored in gene therapy. Mitochondrial gene therapy is no exception, with several polymer-based delivery systems being developed that target mitochondria exclusively [117]. Polymeric systems targeting mitochondria must overcome more barriers than systems that aim to solely cross the cell membrane. It is, therefore, imperative that these transporters are supplemented with ligands that allow mitochondria targeting. To this goal, the most commonly applied ligands are lipophilic cations (e.g., triphenylphosphonium, TPP/Dequalinium, DQA/Rhodamine), MTS/MPP (Figure 4), and DNA and RNA aptamers. These ligands are intended to confer mitochondriotropic properties on polyplexes [117,118].

The lipophilic cation ligands are frequently employed due to their positive charge that facilitates the penetration of polymeric systems through the mitochondrial double membrane. TPP is one of the most used because of its easy incorporation into polymers [119,120]. Marrache et al. demonstrated that systems with TPP were effective in delivering genetic material to mitochondria [121]. These investigators created a PLGA-PEG-TPP system, with PLGA-PEG-OH or PLGA-COOH and demonstrated the internalization of the systems in mitochondria. With this mitochondria-targeted polyplex, they demonstrated the ability to induce immunotherapy by increasing interferon-gamma (IFN-γ) in tumor cells [121].

DQA is another group of lipophilic cations explored for the development of polymer-based carriers toward mitochondria targeting/delivery. The use of DQA in delivery systems has already demonstrated the ability to accumulate in the mitochondrial matrix [122,123]. Another study using DQA was carried out by Faria et al., where a nanocarrier based on polyethylenimine (PEI) was synthesized, with the addition of the TAT peptide and DQA molecules. The PEI–DQA/TAT/pND1 polyplexes display a set of favorable physicochemical properties for cellular uptake and transfection. Moreover, these delivery systems showed greater mitochondria targeting ability depending on the PEI molecular weight and the PEI/pDNA ratio used. The internalization of these PEI–DQA/TAT/pND1 polymeric systems in the mitochondria was demonstrated through confocal microscopy followed by mRNA evaluation (RT-PCR), and quantification of ND1 protein expression (through a specific kit) within the mitochondrial cell extracts [87].

**Figure 5:**
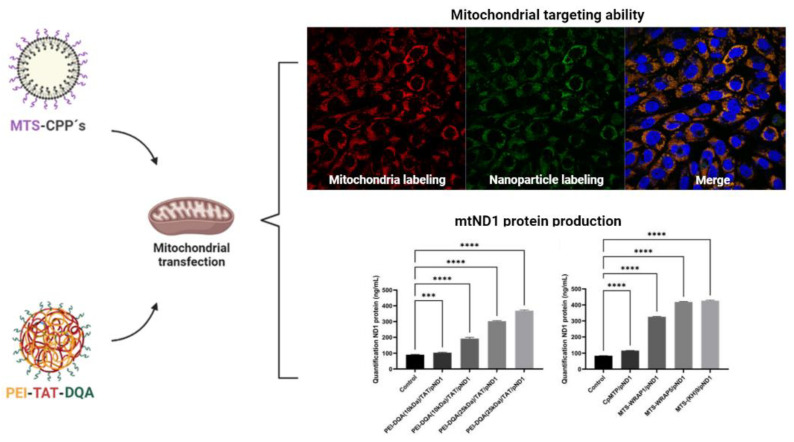
Schematic representation of two strategies/delivery systems developed for mitochondrial gene therapy (MTS-CPPs/pND1 [104] and PEI-TAT-DQA/pND1 [87]) and the most relevant achieved results. The delivery systems demonstrated the ability to target and transfect mitochondria in HeLa cells, a result confirmed by confocal microscopy (Mitochondria were labeled with Mitotracker, the plasmid encapsulated in the systems was labeled with FITC, and nuclei were stained with DAPI). These systems were also able to mediate the delivery of the mitochondrial ND1 gene directly into the mitochondria and the production of the respective protein after transfection of HeLa cells, an achievement demonstrated by the quantification of ND1 using the Elisa kit. Data were analyzed by one-way ANOVA. Data analysis was conducted with GraphPad Prism v.8.01 (GraphPad Software, Inc., San Diego, CA, USA). A *p*-value below 0.05 was considered statistically significant; *** *p* < 0.001 and **** *p* < 0.0001. The images of the results were adapted from [87,104]. Schematic representation created at BioRender.com.

Beyond the development of the PEI-DQA/TAT systems described above, Faria et al. developed also a polymeric system (PEI-PEG) grafted with TPP (PEG-PEI-TPP) [124]. These TPP-polyplexes demonstrate the ability to encapsulate two different plasmids, a plasmid based on the GFP (green fluorescent protein) gene modified to be specifically expressed in mitochondria and a plasmid containing the mitochondrial gene ND1 (mitochondria-encoding NADH dehydrogenase 1 protein). Both plasmid DNA delivery systems were easily internalized into HeLa cells and targeted to mitochondria. Furthermore, mitochondria have been isolated from the cytosol of HeLa cells and a greater accumulation of both GFP and ND1-based plasmid DNA in isolated mitochondria, compared to levels present in the cytosol, was verified. The expression and quantification of ND1 and GFP proteins were demonstrated by these researchers using an Elisa kit, proving that the developed systems promote gene and protein expression, a relevant contribution to mitochondrial gene therapy [124]. The same group also compared PEI-DQA/TAT systems with carriers based on peptides modified with a mitochondrial-targeting sequence (MTS), also designed/developed to create new strategies for mitochondrial gene delivery and expression [87,104]. The peptides used were cell-penetrating peptides (CPP) conjugated to MTS that encapsulated the mitochondrial gene ND1 plasmid DNA (MTS-CPP-pND1) [104]. The created MTS-CPP-pND1 systems show a greater capacity for cellular transfection. This fact contributes to the physicochemical properties exhibited by MTS-CPP-pND1 such as small size and high zeta potential, which resulted in higher cellular uptake, mitochondria targeting, and accumulation when compared to the PEI-DQA/TAT systems. Furthermore, cells transfected with the peptide-based systems demonstrated higher ND1 gene expression, and, consequently, the respective protein was produced to a higher extent compared to those observed with the PEI-DQA/TAT/pND1 systems [87]. These examples of mitochondrial ND1 gene delivery systems are depicted in Figure 5.

#### 2.3.2. Dendrimers

Dendrimers are highly branched polymer-based nanostructures, consisting of a core, branches, and surface groups [125]. The structure and synthesis of dendrimers make this type of delivery system very dynamic and capable of being molded to the intended use. The core–shell structure that characterizes dendrimers allows branch points to be created in their synthesis, added to the vector nucleus, and the shell can be functionalized with ligands that confer specificity. In addition to this synthetic versatility, the fact that it has a core facilitates the encapsulation of molecules inside this type of nanocarrier [126,127].

Mitochondrial-targeted gene therapy using dendrimers as delivery systems was studied by Wang et al. [128]. An ethylenediamine-cored and amine-terminated generation 5 (G5) PAMAM dendrimer to which TPP was added, was synthesized and tested in HeLa and COS-7 cells. The dendrimers under study showed reduced cytotoxicity in both cell lines and demonstrated transfection capacity similar to the commercial transfection reagent Lipofectamine 2000. Functionalization with TPP allowed the transfection of mitochondria, confirmed by the co-localization of the G5-TPP/DNA complexes (FITC-labeled G5-TPP) in the mitochondria, while this co-localization was not shown for the naked G5/DNA systems [128]. Biswas et al. studied a similar system, in which the ligand used was again TPP in combination with the generation 5 poly(amidoamine) (G(5)-D) PAMAM dendrimer. The TPP-modified dendrimers had less cytotoxicity than the TPP-free dendrimers in normal mouse fibroblasts (NIH-3T3). Furthermore, the TPP systems demonstrated specificity for mitochondria and a good ability to internalize into cells. In the work carried out, no molecule was encapsulated, however, the authors report that this system can be exploited for gene delivery into the mitochondria [129].

#### 2.3.3. Lipids

**Figure 6:**
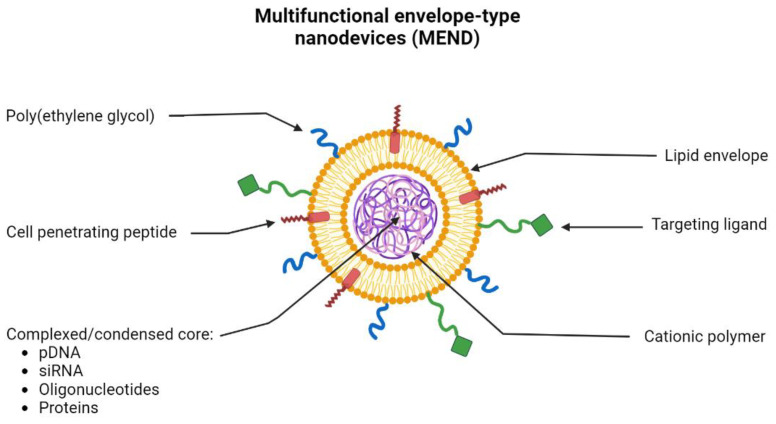
Schematic representation of the morphology and constitution of the multifunctional envelope-type nanodevice (MEND). MEND consists of a lipid envelope that is modified with cell-penetrating peptides (CPP), poly(ethylene glycol) (PEG), and ligands that provide specific targeting. Inside, MEND can encapsulate/condense various therapeutic materials such as DNA, RNA, proteins, etc.

The use of lipids for the creation of gene-delivery nanosystems has been extensively explored for many years. This is due to their properties which allow the formation of a stable nanosystem due to the electrostatic interactions between nucleic acids and lipids but also based on a controlled size of the formed nanoparticles and the protection of the therapeutics inside the core of these transporters [130]. In recent years, the use of multifunctional envelope-type nanodevices (MEND) has been widely explored. This type of lipid-based system allows the encapsulation of different types of molecules such as pDNA, RNA, oligonucleotides, and proteins. Due to the properties of lipids, they can be functionalized with multiple ligands (Figure 6). MEND allows, for example, to add of peptides to its lipid envelope, improving the fusion and internalization of these systems in cells. Ligands can also be added to provide specific targeting to a certain organelle and PEG moieties to improve and prolong the circulation of lipid-based nanoparticles in the bloodstream [131].

Khalil et al. managed to develop a MEND system composed of 1,2-dioleoyl-sn-glycero-3-phosphatidylethanolamine (DOPE) and cholesteryl hemisuccinate (CHEMS) with the CPP R8 coupled at the surface. However, this delivery system did not demonstrate a great capacity to translocate through mitochondrial membranes despite having the ability to transfect dividing cells [132]. Thus, it became relevant to test new lipid compositions that could have greater fusion capacity with the mitochondrial membrane. This subject was explored by Yamada and his coworkers who developed two types of MEND-R8 systems, one with DOPE/SM/STR-R8 (9/2/1) and the other based on DOPE/PA/STR-R8 (9/2/1). Both compositions showed high fusogenic activity with the mitochondrial membrane; however, the lipid systems with SM were considered better for mitochondrial gene therapy because of their lower cytotoxicity [133]. Other researchers also demonstrated that the use of a tetra-lamellar MEND (T-MEND) composed of fusogenic lipids allowed the application of a gene therapy technique directed at mitochondria [134]. A study in which two innovative MEND systems (β-MEND (DC-Chol/EPC/SM (3/4/3)) and RP/β-MEND (DC-Chol/EPC/SM/Chol-RP (3/4/3/0.25)), demonstrated that these nanocarriers can efficiently deliver genetic material directly into the mitochondria of H9c2 cells, a mouse cardiomyoblast cell line. These two systems revealed the ability to target mitochondria and they are suitable for gene delivery, therefore, instigating further studies concerning mitochondrial gene therapy against cardiomyopathies [135].

Another type of lipid-based compound widely used for the delivery of nucleic acids to mitochondria is DQAsomes. DQAsomes are positively charged lipid vesicles that allow the encapsulation of nucleic acids and display an affinity for binding to the membrane of mitochondria [100]. These complexes can escape from the endosomes without losing the encapsulated genetic material and carry the therapeutic payload next to the mitochondria or inside the mitochondrial matrix, depending on its composition [136,137]. To overcome some difficulties that the DQAsome systems may present regarding cellular internalization, the incorporation of different lipids forming the DQA80s was tested. The DQA80s system has a higher transfection efficiency when compared to the DQAsome; however, it delivers most of the genes around the mitochondria and not inside [136].

MITO-Porters are liposome-based delivery systems that enable the delivery to mitochondria of any type of material that it encapsulates, regardless of properties and size. Its membrane fusion mechanism responsible for the targeted delivery to mitochondria is divided into three phases. In the first phase, the systems must enter the cytosol, then their targeting to the mitochondria and the pathway inside the cell has to be regulated, and finally the regulation of membrane fusion for delivery to the mitochondria [138]. In this sense, Yamada and Harashima created a high-density octaarginine (R8)-modified MITO-Porter and demonstrated the ability of these systems to internalize into mitochondria. The presence of the cationic peptide R8 not only allowed binding to mitochondria that are negatively charged in their membrane but also facilitated the internalization of these systems via macropinocytosis in the cytoplasm. These systems have demonstrated the capacity to deliver macromolecules such as nucleic acids and proteins directly to mitochondria [139]. Kawamura et al. developed a MITO-Porter capable of delivering nucleic acids to mitochondria with its functioning altered by a heteroplasmic G625A mutation in mtDNA tRNA^Phe^ [140]. Once again, the MITO-Porter has been modified on its surface with R8, which makes it possible to escape from the macropinosomes to the cytosol. Once in the cytosol, MITO-Porter binds by electrostatic interactions to the mitochondrial membrane and fusion occurs between the delivery system and the mitochondria. The transfection capacity of this system was demonstrated by the PCR technique, where cells transfected with MITO-Porter led to pre-WT-tRNA^Phe^. Moreover, researchers observed that this effect lasted up to 8 days after the transfection. Furthermore, cells transfected with this liposome-based system demonstrated a replenishment of cellular respiration levels [140]. Another work also demonstrated the use of a MITO-Porter (ASO [COX2]/PEI) that managed to encapsulate and deliver an antisense RNA oligonucleotide (ASO) to the mitochondria, leading to the silencing of the target gene, and consequently, to the reduction of corresponding mRNA levels within the mitochondria [141]. An interesting study used a MITO-Porter in which the surface was modified with a mitochondrial RNA aptamer and the KALA peptide and inside it was encapsulated a DNA vector (pCMV-mtLuc (CGG)), specific to be transcribed in the mitochondria. Fibroblasts from a patient with mitochondrial dysfunction were transfected with the developed system and the transfected cells were found to have higher mitochondrial luciferase levels compared to untransfected cells [142].

#### 2.3.4. Inorganic Nanoparticles

Rhodamine is a fluorescent compound with affinity to mitochondria, which facilitates the tracking of internalization into cells, and it has been applied as a probe for mitochondrial membrane potential [143]. This property of rhodamine has been explored for the targeting and gene delivery to mitochondria, as described by Santos et al. [144]. The researchers developed plasmid DNA-based rhodamine nanoparticles, using CaCl_2_, Na_2_CO_3,_ and cellulose, in a co-precipitation method, to formulate the nano-systems. The inorganic compounds were able to promote the encapsulation of three different plasmids and internalize them in several types of cell lines, also demonstrating the ability to target mitochondria, confirmed by confocal microscopy [144]. The team of Costa formulated CaCO_3_-pDNA-Rho123 nanoparticles using the same co-precipitation method [145]. The vectors demonstrated the ability to encapsulate a plasmid containing the GFP gene, formulating stable nanoparticles of suitable size and surface charge for cell transfection. These delivery systems have demonstrated transfection capability into both normal and tumoral cells. Images obtained by two- and three-dimensional fluorescence confocal microscopy confirmed the targeting of these vectors to mitochondria in both fibroblasts and HeLa cells. This targeting was demonstrated by rhodamine fluorescence and by the expression of the GFP protein. To confirm the affinity of the systems for mitochondria, after cellular transfection, mitochondria were separated from the cytosolic fraction, verifying greater accumulation of nanoparticles in isolated mitochondria compared to the cytosol fraction, through quantification of rhodamine fluorescence [145]. Costa et al. also created calcium carbonate delivery systems capable of encapsulating p53 and ND1-GFP plasmids [146]. The nanoparticles formulated with the compound [16]phenN_2_ showed an affinity for mitochondria. The fluorescence of [16]phenN_2_ in isolated mitochondria was quite high when compared to the fluorescence of this compound in the cytosol or in lysosomes, where fluorescence was residual. Mitochondrial targeting of systems with [16]phenN_2_ was further reinforced when plasmids were replaced by BSA, which resulted in an accumulation of BSA within the mitochondria of cells transfected with these nanoparticles, while this accumulation did not occur in the cytosol and the lysosome [146].

### 2.4. Strategies to Be Further Explored for Progress in Mitochondrial Gene Therapy

Mitochondrial gene therapy is a recent area of research and therefore there are still many possibilities that can be explored. The delivery system design is a fundamental step for mitochondria targeting and transfection sucess. Delivery vectors that have been specifically developed to deliver therapeutic molecules such as drugs and antioxidants to mitochondria are a good starting point. Thus, we intend to present some delivery systems already studied that offer great potential to be explored in mitochondrial gene therapy.

#### 2.4.1. Dendrigraftpoly-L-lysines Based Delivery Systems

Biocompatible dendritic poly(L-lysine) (DGL) is a promising carrier for targeted drug/DNA delivery [147]. DGL is becoming one of the most versatile nanoscale drug/DNA carriers due to its highly branched 3D architecture containing an initiator core, several inner layers composed of repeating units, and many outer amino groups [148]. Compared with traditional nanocarriers, self-organized DGL nanoparticles are vastly superior in targeted therapy at the subcellular level due to their small size and their surface modifications (PEGylation and targeted ligands). In particular, DGL nanoparticles (DGL NPs) have a greater ability to facilitate intracellular internalization via endocytosis, and then in the endosomes, they act as proton-sponges to induce endo-lysosomal escape by osmotic swelling [149]. These properties of DGL NPs make them attractive nanocarriers for the construction of targeted drug delivery systems to mitochondria.

Chen et al. synthesized a delivery system based on dendrigraftpoly-L-lysines (DGL) with incorporated doxorubicin (Dox) that was intercalated into a DNA duplex containing an ATP aptamer. In addition, a Cytochrome C aptamer, and a nucleolin-specific binding aptamer, AS1411, were added to the nanoparticles (Dox/Mito-DGL). This study demonstrated that this dual system showed targeting and accumulation in the mitochondria, the therapeutic agent was released and allowed to improve the efficacy in multidrug-resistant cancer cells [150].

#### 2.4.2. ROS-Responsive PLGA-Based Delivery Systems

The ROS-responsive berberine PLGA-based nanocarriers (BPseP) were designed with the idea that tissue areas with high inflammation and high ROS concentration could irritate the cleavage of the mPEG–Se–Se–PLGA amphiphilic co-polymers and berberine will be released to produce higher ROS, which further facilitates the collapse of micelles [151]. These specific ROS-responsive delivery systems were studied to develop a therapy for rheumatoid arthritis (RA), an autoimmune disease with no cure. Indeed, Fan and co-workers demonstrated that in RA fibroblasts the drug uptake was ten times higher compared to what happened in non-treated fibroblasts. The therapeutic effect obtained through mitochondrial targeting and drug release in the inflationary zone was shown by the inhibition of cell proliferation and elimination of lipogenesis, improving the therapeutic efficacy of AR [151].

Another example of ROS-responsive nanocarriers was described by Zhou et al. creating lipid-polymer hybrid nanoparticles (LPNPs) composed of a redox-responsive polymer (DLPE-S-S-mPEG4000), an amphiphilic polymer with TPP (C18-PEG2000-TPP), and PLGA. This system can accumulate in mitochondria and because of its cationic properties, it can be an excellent system for encapsulating nucleic acids [152].

Nanoparticles composed of cerium oxide (CeO_2_-NPs; <5 nm) are known to remove ROS via superoxide dismutase (SOD) mimetics and remove OH via redox reactions using their Ce ^3+^ sites, while the Ce ^4+^ sites are responsible for H_2_O_2_ oxidation via catalase (CAT) mimetics [153]. CeO_2_-NPs were modified with triphenylphosphine, followed by coating with ROS-responsive organic PLGA-polymer to obtain ROS-responsive nanocarriers to treat acute kidney injury (AKI) [154]. By loading atorvastatin, which has been reported to have an ameliorative effect on acute kidney injury, the authors demonstrate an increased therapeutic effect of the CeO_2_-NPs through synergistically anti-inflammatory and antioxidant effects.

In conclusion, the rapid development of ROS-sensitive nanocarriers has shown their applications in many biological systems. However, even if significant progress was made some, steps still need optimization such as the controlled drug release and the in vivo therapeutic effects of these nanocarriers to guarantee their commercialization.

#### 2.4.3. MITO-Porter-Based Systems

MITO-Porter is a liposome-based carrier that delivers macromolecules efficiently to the cytoplasm, as well as to mitochondria. In 2008, Yamada et al. coated the MITO-Porter surface with high-density octaarginine (R8) to deliver green fluorescence protein (GFP) to rat-liver mitochondria [138]. Membrane fusion occurs by two extremely fusogenic lipid compositions: sphingomyelin (SM) and phosphatidic acid (PA) resulting in macropinocytosis instead of clathrin-mediated endocytosis, which allowed particles to enter the cell without being damaged. The MITO-Porter was further optimized by the S2 peptide (Dmt-d-Arg-FK-Dmt-d-Arg-FK-NH2) instead of the R8 showing a high mitochondrial targeting activity with less cellular toxicity [155].

Another study demonstrated interesting results when using a MITO-Porter nanosystem that demonstrated the ability to encapsulate and deliver the antioxidant CoQ10 in the liver tissues of mouse models. By confocal laser scanning microscopy, they confirmed the accumulation of this antioxidant in the mitochondria of liver cells from the transfected mice. They also verified that the presence of CoQ10 led to a decrease in ROS, which was confirmed by the decrease in ALT levels in transfected hepatic tissues [156].

#### 2.4.4. Upgraded TPP-Based Systems

As detailed in Section 2.3.1 and Section 2.3.2, TPP moieties were added to different nanocarriers to optimize their mitochondriotropic properties.

For example, a study carried out by Sharma and the team compared a dendrimer in which TPP molecules (TPP-D-Cy5) were coupled with the same dendrimer without TPP (D-Cy5). They showed that although these systems do not have a fully specific targeting for mitochondria, systems with TPP had more affinity for mitochondria. However, D-Cy5 dendrimers were also able to accumulate in mitochondria but in reduced numbers. The authors of this study also demonstrated that cells transfected with TPP-D-Cy5 showed improvement in levels and markers of oxidative stress [157]. Li and co-workers developed a micelle where they coupled TPP (PEG-AIE-TPP), a nanocarrier synthesized to be targeted to the mitochondria and used in the treatment of cancer due to the pH sensitivity [158]. These investigators demonstrated that these micelles selectively accumulated in mitochondria through the conjugation of a fluorogenic that produced an aggregation-induced emission (AIE) effect. Through confocal microscopy, it was possible to observe the superposition of this signal produced by PEG-AIE-TPP nanoparticles with the labeling of mitochondria. These micelles were used in MCF-7 tumor-bearing mice, where they were observed to preferentially accumulate in the tumor both in vivo and ex vivo [158].

Yang et al. developed a micellar nanosystem that aims to deliver the antioxidant resveratrol to neuronal mitochondria, trying to contribute with a strategy to combat Alzheimer’s disease [159]. These formulated nanoparticles are composed of PEG-b-poly(l-lactide) (PLA), grafted with the brain neuron specific binding peptide C3 and the mitochondrial targeting TPP. This study demonstrated the ability of these vectors to be delivered specifically to the mitochondria of central neurons, a targeting provided by C3 that functions as a neural cell adhesion molecule (NCAM) and by TPP [159]. Zhou et al. created nanoparticles with a redox-responsive polymer (DLPE-S-S-mPEG4000), an amphiphilic polymer with TPP (C18-PEG2000-TPP), and PLGA. This system can accumulate in mitochondria and because of its cationic properties, it can be an excellent system for encapsulating nucleic acids [152].

Chitosan/sodium alginate nanoparticles to which a TPP^+^-g-CS polymer was added were developed by Arafa et al. [160]. These SAL/TPP^+^-g-CS vectors demonstrated the ability to encapsulate therapeutic material through electrostatic interactions, forming stable nanoparticles with reduced and homogeneous size. Due to the presence of TPP in the polymer used in the outer coating, these nanocarriers demonstrated targeting to the mitochondria. In vitro studies have shown that these mitotropic systems have low toxicity and induce a therapeutic response in tumor cells. These results were proven in vivo, where the profile of reduced toxicity and increased antitumor activity in tissues transfected with these systems was demonstrated [160].

However, recent studies have revealed that the TPP^+^ moiety has detrimental effects on mitochondrial bioenergetics such as increasing proton leak and uncoupling mitochondrial oxidative phosphorylation (OXPHOS). Therefore, efforts were performed to identify TPP^+^ derivatives associated with mitochondrial uptake without OXPHOS decoupling to develop improved TPP^+^ derivatives for targeting cargos/carriers to mitochondria [161].

Altogether, these systems mentioned above, among others [162], have demonstrated the ability to transfect and internalize into mitochondria and release their therapeutic content. Therefore, this skill can be deeply investigated for the design and development of targeted mitochondrial genes-based delivery systems. Future studies should focus on the conception of novel nanosystems able to efficiently encapsulate mitochondrial genetic material and the optimization of their physicochemical properties towards effective mitochondria transfection, gene, and protein expression. This effort will certainly constitute a step ahead in mitochondrial gene therapy, a great contribution to the treatment of mtDNA diseases.

## 3. Concluding Remarks/Future Perspectives

Mitochondrial diseases can have different origins and mostly derive from genetic mutations, both in mtDNA and in nuclear DNA. This heterogeneity complicates the development of drugs that can act at the origin of mitochondrial dysfunctions. Currently, the therapeutic options available on the market serve only to mitigate symptoms aiming to improve the quality of life of patients, none of which provide effective treatment or cure. Therefore, there is a crescent demand for new strategies to tackle this problem at its source. Mitochondrial gene therapy emerges as a promising strategy, as it can replace or block the effect of genetic mutations that induce mitochondrial diseases. Various types of transporters can be used to deliver therapeutic genetic material to mitochondria to reestablish the normal activity of this organelle. Among the most used compounds in the development of mitochondrial gene delivery systems are polymers, lipids, and MITO-Porters, among others. Although there are already studies with a wide choice of nanocarriers, this area of research is still very recent and needs more studies (in vitro and in vivo) to provide a valid option for patients suffering from mtDNA mitochondrial diseases. Taking advantage of the work already done for the delivery of other types of therapeutic molecules specifically for mitochondria, some of the mentioned delivery systems can be adapted and studied towards mitochondrial gene therapy. Many of the delivery systems can be modified both in their composition and in their structure to promote the encapsulation and stability of mitochondrial genetic content while improving mitochondria targeting and overcoming the various barriers vectors must face in cellular uptake and transfection processes. In the future, transporters showing good performance in gene therapy should be improved with ligands that facilitate not only their penetration and cellular internalization but also provide their specific targeting to mitochondria. This joint effort, hopefully, will lead to great advances in the design/development of suitable mitochondria-targeted delivery systems able to promote effective and sustainable mitochondrial transgene expression.

## Figures and Tables

**Table 1:** Summary of mitochondrial genes affected by mutations and the associated diseases.

Mutated Mitochondrial Gene	Associated Disease	Reference
ND1	Type II diabetes	[44]
	Leber’s hereditary optic neuropathy (LHON)	[46]
	Leigh’s syndrome	[66]
	Progressive cardiomyopathy	[67]
	Neurodevelopmental delay	[68]
	Sensorineural hearing loss	
	Renal cancer	[57]
	Thyroid carcinoma	[62]
	Oncocytic pituitary adenoma	[64]
	Breast carcinoma	[65]
ND3	Type II diabetes	[45]
	Parkinson’s disease	[52]
ND5	Invasive ductal breast carcinoma	[62]
ATP6	Type II diabetes	[43]
	Mitochondrial encephalomyopathies	[49]
	Alzheimer’s disease	[53]
	Hurthle cell thyroid carcinoma	[58]
	Breast cancer	[63]
Desmin	Desmin myopathies	[48]
	Skeletal myopathy	
C9orf72/TARDBP	Frontotemporal dementia	[50]
	Amyotrophic lateral sclerosis	
COXIII	Invasive lobular breast carcinoma	[62]

## Data Availability

Not applicable.
